# Candidate protein biomarkers in pancreatic neuroendocrine neoplasms grade 3

**DOI:** 10.1038/s41598-020-67670-7

**Published:** 2020-06-30

**Authors:** Abir Salwa Ali, Aurel Perren, Cecilia Lindskog, Staffan Welin, Halfdan Sorbye, Malin Grönberg, Eva Tiensuu Janson

**Affiliations:** 1 Department of Medical Sciences, Section of Endocrine Oncology, Uppsala University, Rudbecklaboratoriet, hus R3, vån 2, Dag Hammarskjölds väg 20, 752 85 Uppsala, Sweden; 2 Department of Pathology, University of Bern, Bern, Switzerland; 3 Department of Immunology, Genetics and Pathology, Uppsala University, Uppsala, Sweden; 4 Department of Oncology, Haukeland University Hospital, Bergen, Norway; 5 Department of Clinical Science, University of Bergen, Bergen, Norway

**Keywords:** Neuroendocrine cancer, Tumour biomarkers

## Abstract

Pancreatic neuroendocrine neoplasms (PanNENs) are rare tumours that compose 1–2% of all pancreatic tumours. Patients with metastatic grade 3 neoplasia are usually treated with chemotherapy but have a poor progression-free and overall survival. According to the WHO 2017 classification, they are divided into neuroendocrine tumours (NETs) G3 and neuroendocrine carcinomas (NECs). Despite the new classification, new diagnostic and prognostic biomarkers are needed to sub-categorise the patients and to help guide therapy decisions. Blood from 42 patients and 42 healthy controls were screened for the presence of 92 proteins with the Immuno-Oncology panel using the Proximity Extension Assay provided by Olink Biosciences. Immunohistochemical staining of FAS ligand (FASLG) was performed on 16 patient tumour specimens using a commercial antibody. Fifty-four out of 87 evaluable proteins differed significantly in concentration between blood from patients and blood from healthy controls. FASLG was the only protein for which the concentration in blood was significantly lower in patients compared to controls and the levels correlated negatively to Ki-67 index. Seven of 14 evaluable PanNEN G3 specimens showed FASLG immunoreactivity in the tumour cells while there was scattered immunoreactivity in immune cells. Positive FASLG immunoreactivity correlated to well-differentiated morphology. FASLG concentration in blood was significantly lower in patients with pancreatic NENs G3 compared to controls, and the expression in tumour tissue was variable. Furthermore, FASLG was negatively correlated to Ki-67 and was more frequently expressed in well-differentiated tumours. Taken together, these results may suggest a role of FASLG in PanNENs.

## Introduction

Pancreatic neuroendocrine neoplasms (PanNENs) are rare tumours and comprise approximately 1–2% of tumours in the pancreas, with a rising incidence in the last decade^1,2^. There is no evidence of differences in incidence between races, gender or geographical location^3^. To determine the neuroendocrine feature of tumours originating from the pancreas, the biomarkers chromogranin A (CgA) and synaptophysin (Syn) are used for immunohistochemical staining^4^.

The histopathological differentiation of tumours into well- or poorly differentiated has in recent years become an important aspect for the diagnosis, therapy choice and outcome assessment of NENs. No difference among PanNENs has been seen concerning location at head versus body/tail and tumour size. Recent publications has shown the presence of a group of tumours that are well-differentiated, but with Ki-67 index > 20%. In these studies, well-differentiated tumours were illustrated to have a better prognosis than poorly differentiated NENs G3^5–8^. However, there are not many head to head comparisons of these two disease entities. In 2017, an update of the WHO classification was published with regards to PanNENs, where both proliferation, genetic background and differentiation are factors taken into consideration. Here, PanNENs that belong to the G3 group are defined as either well-differentiated with a Ki-67 > 20%, pancreatic neuroendocrine tumour grade 3 (PanNET G3) or as poorly differentiated pancreatic neuroendocrine carcinoma (PanNEC) with Ki-67 > 20%. The latter is also divided into small cell and large cell morphology^9^.

Treatments of PanNENs are different, pertaining their proliferation and differentiation. Metastatic PanNECs are traditionally treated with platinum based chemotherapy in combination with etoposide^8,10–12^ while PanNET G3 may be treated more like PanNETs G2 with surgery, peptide receptor radionucleotide therapy, everolimus, temozolomide/capecitabine or sunitinib^8,13^. Genetically, PanNENs differ with regards to grade and differentiation. A study has shown that PanNETs G3 commonly harbour mutations in the *MEN1, ATRX* and *DAXX* genes while PanNECs have alterations in *TP53* and *RB1*^14^. However, there are very few studies that address the question of how these two entities should be handled in the clinic.

Until now, very little is known about the presence of biomarkers in blood in PanNEN G3 patients^15^ and if there are any biomarkers that might be useful for diagnosis, prediction of therapy or prognosis. In the interest of understanding PanNENs G3 and their complexity, we need to understand their molecular composition. Detection of protein biomarkers in blood is a commonly used way to identify such molecules, and methods in the area have advanced. A recently developed technique is the Proximity Extension Assay (PEA), a sensitive and specific method, which uses a DNA polymerisation step for protein detection^16^.

The aim of this study was to screen for biomarkers of clinical interest in blood from patients with PanNENs G3 and compare the concentrations with healthy controls using the Olink PEA technique.

## Results

### Biomarker expression in blood

**Figure 1:**
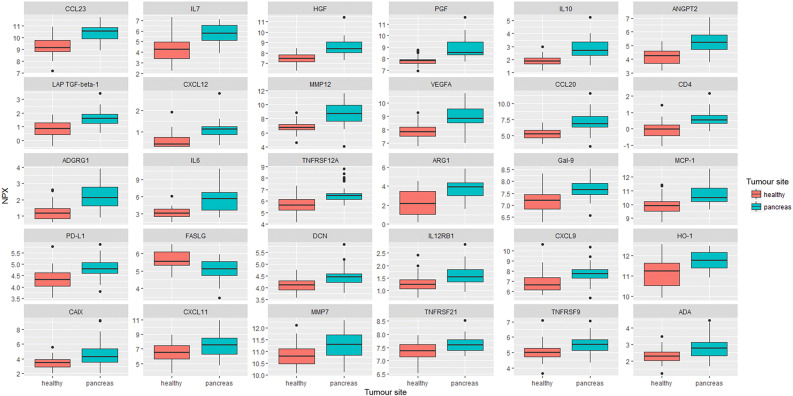
Boxplots of serum proteins in healthy controls and PanNEN G3 patients, for the 30 most statistically significant proteins.

Patient characteristics are summarised in Table 1. In total, 79 out of 84 (94%) blood samples where included in the statistical analysis, five patient blood samples did not meet the quality control guidelines of the Olink Biosciences lab. Proteins with a lower detectability than 15% were removed, resulting in 87 out of 92 (95%) proteins included in the statistical analysis. Fifty-four (62%) proteins had significantly different blood levels when comparing patients and controls (*p *value < 0.05). All were proteins expressed in higher concentrations in tumour samples than healthy controls, except for FASLG. Blood samples from PanNEN patients had significantly lower concentrations of FASLG than blood samples from healthy controls (Fig. 1).

**Table 1:** Clinicopathological characteristics of patients included in the Olink assay.

Variables	Median (range)
Age (years)	59 (27–80)

	*n* (%)
**Sex**	
Male	27 (64)
Female	15 (36)
**Chromogranin A immunohistochemistry**	
Positive	38 (90)
Negative	4 (10)
**Synaptophysin Immunohistochemistry**	
Positive	40 (95)
Negative	2 (5)
**Ki-67**	
< 55%	30 (71)
> 55%	12 (29)
**Response according to RECIST criteria**	
Progressive disease	14 (33)
Stable disease	8 (19)
Partial response	15 (36)
Complete response	0 (0)
Missing data	5 (12)

**Figure 2:**
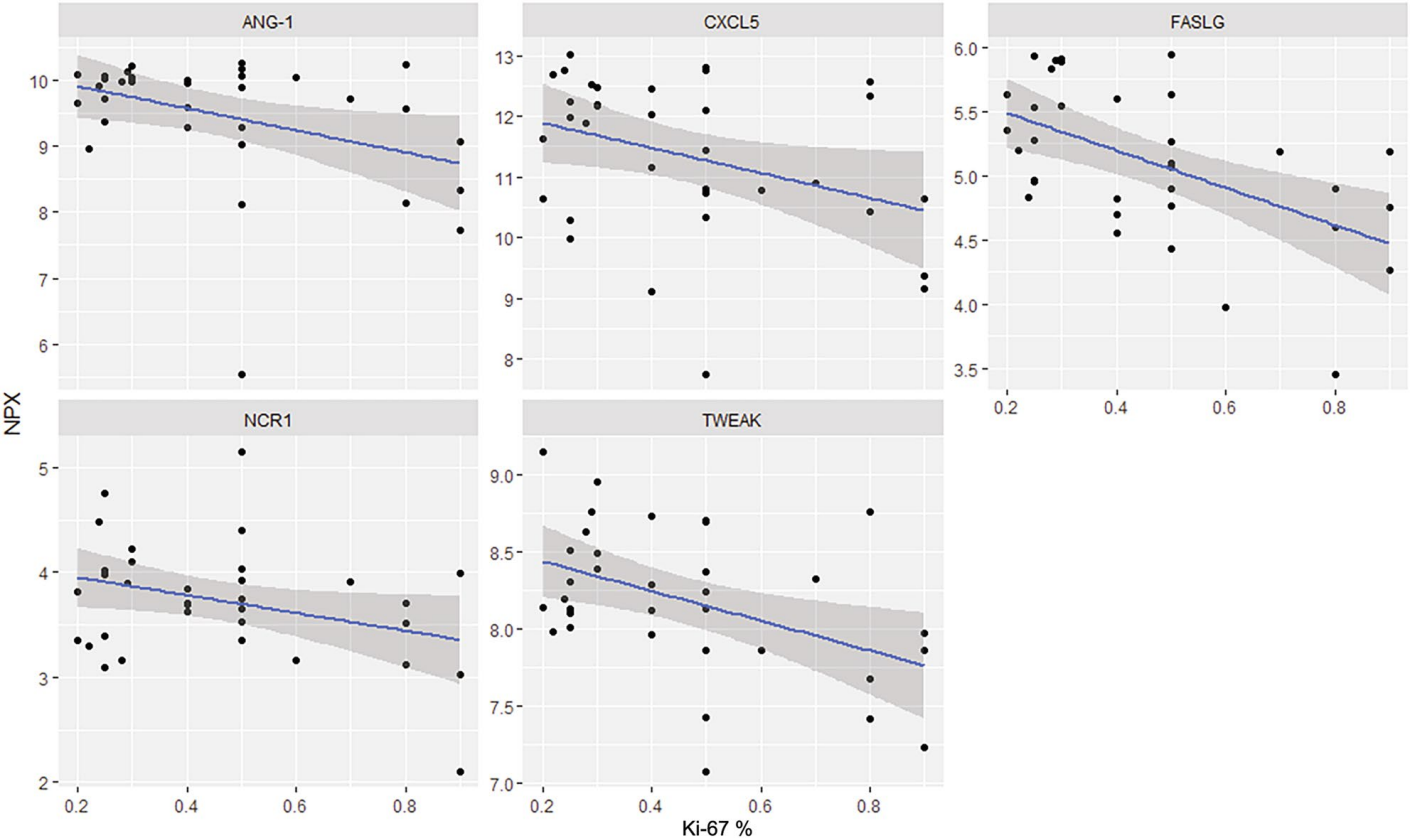
Linear regression plots for five proteins and their correlation to Ki-67 index. On the x-axis is Normalized Protein eXpression (NPX), a measurement for protein concentration, and on the y-axis is the Ki-67 index in percentage. All *p* values < 0.05, unadjusted.

In a regression analysis to associate blood proteins with Ki-67 cut off levels of < 55% and > 55%, none of the proteins was significantly correlated to Ki-67, when adjusted for multiple testing with the Benjamini–Hochberg approach. Five proteins [Angiopoietin-1 (ANGPT1), CXC chemokine ligand 5 (CXCL5), FASLG, Natural cytotoxicity triggering receptor 1 (NCR1) and Tumour necrosis factor-like weak inducer of apoptosis (TWEAK)] correlated negatively to Ki-67 using unadjusted *p *values < 0.05 (Fig. 2), with FASLG being the protein of highest interest for G3 PanNENs.

There were no proteins associated to earlier death, neither when adjusted for long time and short time survivors (data not shown).

Correlation of protein levels to treatment response according to the RECIST 1.0 criteria resulted in six proteins [Chemokine (C–C motif) ligand 23 (CCL23), Chemokine (C–C motif) ligand 8 (CCL8), Chemokine (C–C motif) ligand 4 (CCL4), Class I-restricted T cell-associated molecule (CRTAM), Chemokine (C–X–C motif) ligand 1 (CXCL1) and Interleukin-18 (IL18)] that differed significantly between responders and non-responders, although only in the unadjusted assay. The proteins did not seem to differ significantly when corrected for multiple testing.

### Immunohistochemical analysis of FASLG

**Figure 3:**
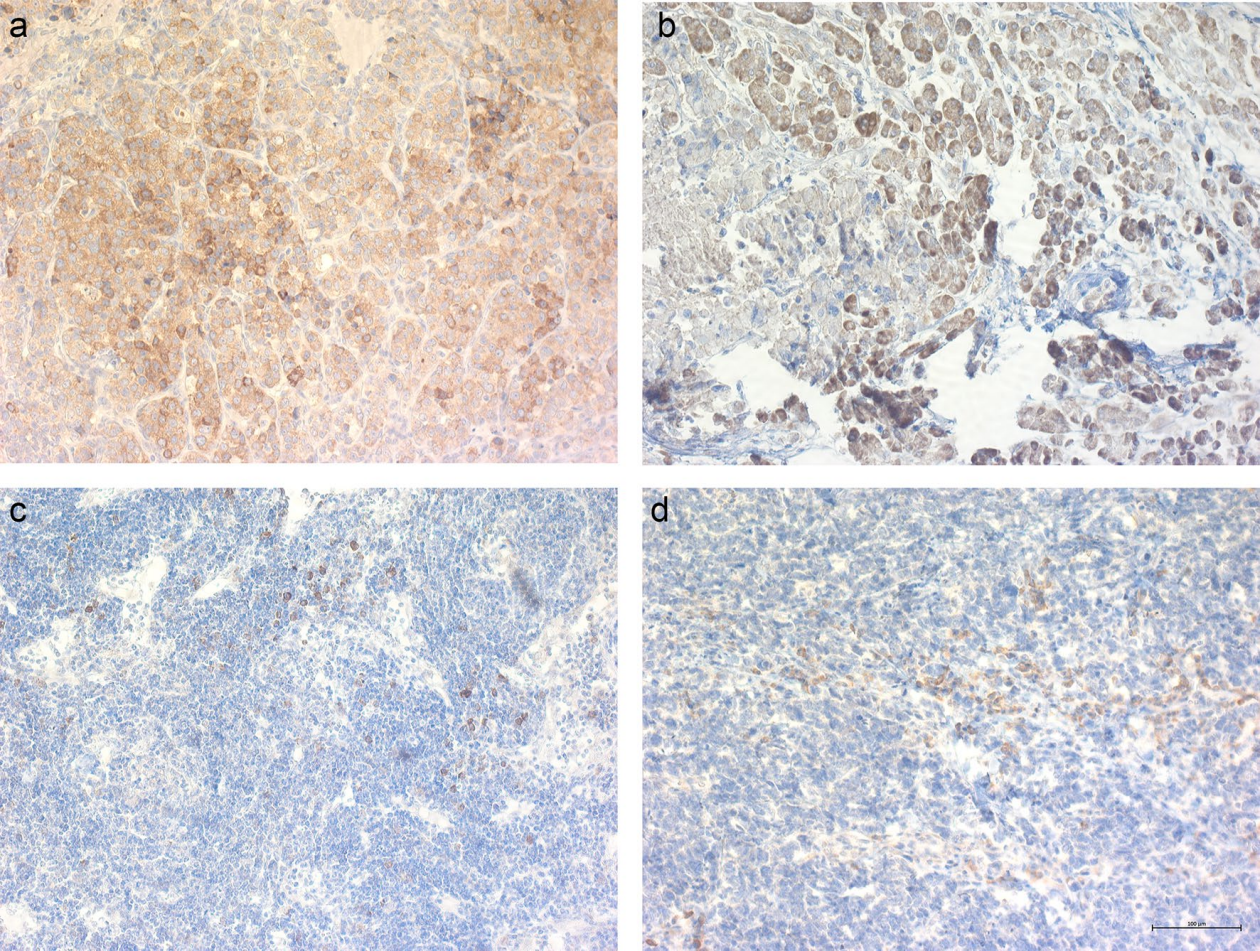
PanNEN G3 immunostained for FASLG. (**a**), (**b**) and (**c**) FASLG immunoreactivity in membrane of tumour cells. (**d**) FASLG immunoreactive immune cells infiltrating a non-immunoreactive tumour. Scale bar 100 µm.

FASLG was the only protein that was present in lower levels in patients than in healthy controls. To further investigate this, immunohistochemical staining of FASLG was performed. Of the 16 tumour samples included in the immunohistochemical analysis, one patient’s tumour sample was excluded due to poor sample quality and another due to missing clinical data, resulting in a total of 14 specimens. Among these samples, 10 had poorly differentiated morphology and four had well-differentiated morphology. Seven out of 14 (50%) specimens were immunoreactive for FASLG in the tumour cells. All FASLG immunoreactive tumour specimens exhibited an organoid structure, with diffuse cytoplasmic and a more evident peripheral membranous staining pattern. Representative images of the immunostainings are shown in Fig. 3. Out of the seven specimens with immunoreactive tumour cells, three were from poorly differentiated and four from well-differentiated tumours, data presented in Table 2.

**Table 2:** FASLG expression in well- and poorly differentiated tumour samples.

Patient	Differentiation	FASLG immunoreactivity	FASLG H-score	Ki-67 index (%)
1	Well-differentiated	Positive in tumour cells	50	25
2	Well-differentiated	Positive in tumour cells	200	50
3	Well-differentiated	Positive in tumour cells	80	80
4	Well-differentiated	Positive in tumour cells	160	80
5	Poorly differentiated	Positive in tumour cells	200	30
6	Poorly differentiated	Positive in tumour cells	100	50
7	Poorly differentiated	Positive in tumour cells	50	80
8	Poorly differentiated	Negative in tumour cells	0	20
9	Poorly differentiated	Negative in tumour cells	0	30
10	Poorly differentiated	Negative in tumour cells	0	40
11	Poorly differentiated	Negative in tumour cells	0	50
12	Poorly differentiated	Negative in tumour cells	0	50
13	Poorly differentiated	Negative in tumour cells	0	75
14	Poorly differentiated	Negative in tumour cells	0	90

H-score; The intensity of staining (graded 0 for negative, 1 for intermediate and 2 for strong), multiplied by the percentage of stained tumour cells.

The immunostaining on the positive control (tonsil tissue) resulted in membranous and cytoplasmic immunoreactivity in a subset of non-germinal centre cells as reported in the Human Protein Atlas (https://www.proteinatlas.org) (data not shown).

Statistical analysis showed that protein expression of FASLG was associated with differentiation. In a chi-square test, well-differentiated tumours were significantly more immunoreactive for FASLG compared to poorly differentiated (*p* value = 0.018).

## Discussion

In this study, we aimed to identify proteins in blood from PanNEN G3 patients that differed in concentration compared to healthy controls. The Olink Bioscience chips revealed that 54 of 87 proteins differed significantly and amongst these proteins were many interleukins and cytokines. All 54 proteins showed higher blood concentrations in tumour samples compared to samples from healthy controls, except for FASLG, which was reversed with lower concentrations in patients. FASLG is a protein mainly present on the membrane of cytotoxic T lymphocytes. It belongs to the tumour necrosis factor (TNF) family, and binds to its receptor FAS on different cells and tissues, including tumour cells and immune cells, i.e. both FASLG and FAS can be expressed by the cytotoxic T lymphocytes^17,18^. FASLG is one of the key components expressed in cytotoxic T lymphocytes^19,20^ and is predominantly found in activated T lymphocytes and natural killer cells (NK-cells)^21^. Binding of FAS to FASLG triggers an apoptotic reaction via conversion of 8 zymogen into an active form^22^. Both FAS and FASLG are important in cancer cell immunity as they have been seen to have both tumorigenic and tumour suppressive roles^23,24^. Circulating FASLG in cancer has been seen before, where a correlation with thyroid cancer recurrence was demonstrated. This suggests that FASLG may be used as a biomarker for disease recurrence^25^. It is unclear what the exact clinical relevance of FASLG in NENs is, but in this cohort, FASLG seems to be present in lower concentrations in blood from patients compared to controls. Interestingly, FASLG correlated negatively to Ki-67, in an unadjusted test. The higher Ki-67 index among the patients, the less concentrations of FASLG were seen in tumour samples, i.e. FASLG seems to be less present in patients with presumed poorer prognosis^26^.

The FAS–FASLG interaction has been shown to be a mechanism by which tumours can escape the immune system. An immune cell that encounters a cell with FASLG will undergo apoptosis. Tumour cells have adapted the expression of FASLG on their cell membranes to deactivate immune cells and hence, eliminate them from entering the tumour microenvironment^27^. Many studies have reported that FAS expression is aberrant in various pancreatic tumours while FASLG is seen in both normal pancreatic cells and pancreatic neoplasia. This may suggest that the aberration of FAS in tumours may be yielding the immune-evasion mechanism and that FASLG expression on tumour cells results in the inactivation of killer T cells and hence becoming resistant to FAS–FASLG mediated apoptosis^28–30^. In a study on autoimmune diabetes, in non-obese mice, soluble FASLG did not result in apoptosis of beta cells and was not a risk factor for developing autoimmune diabetes^31^. FASLG in sera is reported to be increased in many cancers eluding to the mechanism of FASLG as a “counterattack” protein in different tumours. In some studies it has been claimed that membrane bound FASLG induces apoptosis while soluble FASLG promotes tumour cell survival^32,33^. Nevertheless, too little has been studied on the differences between soluble (i.e. sera concentrations of FASLG) and tissue expressed FASLG and which of the two that may be the driving factor for tumour progression^24^.

In our study the immunohistochemical results showed that 7/14 of the PanNEN G3 tumours expressed FASLG in the tumour cells. A correlation between FASLG and differentiation was seen in the immunohistochemical results where well-differentiated tumours were significantly more immunoreactive for FASLG compared to poorly differentiated tumours.

The clinical relevance of FASLG protein expression in tumour cells is not fully understood. The discovery of FASLG and its mechanism in apoptosis in the immune system has given rise to the possibility of a counter-attack mechanism adapted by tumours^34^. Studies have shown that activated T-cells can be de-activated by FASLG expressed on tumour cells^35^. Overexpression of FASLG by tumour cells has also been demonstrated to decrease the number of tumour infiltrating immune cells and increase apoptosis of immune cells^36,37^. A study showed that ovarian carcinomas that expressed FASLG had a significantly poorer prognosis^38^, however contradictory results have also been reported where one study suggests that apoptosis of colon tumour cells and better prognosis, is independent of FASLG expression^39^. Another report with colon cancer patients showed that FASLG expression is not correlated to any clinicopathological parameters i.e. FASLG would not be an immunoprotective factor^40^. In our cohort, FASLG was inversely correlated to Ki-67 where patients with highly proliferative tumours had significantly lower blood concentrations of FASLG. Also, patients with well-differentiated tumours had a significantly higher expression of FASLG compared to patients with poorly differentiated tumours, 100% vs. 30% of tumours being positive. Differentiation and Ki-67 are two well-known factors in disease progression for PanNENs G3, and the association of to both these factors may therefore be of interest for further elucidation.

The occurrences of mutations in *TP53* and *RB1* are common in PanNECs distinguishing them from their well-differentiated counterparts^14^. PanNECs have been shown to harbour mutations in *TP53* and *RB1* while 20–26% of PanNETs harboured mutations in *ATRX* and *DAXX*, a mutation that does not occur in PanNECs^41^. Interestingly, p53 plays a role in the FAS–FASLG apoptosis axis. p53 can induce apoptosis through an intrinsic pathway and an extrinsic pathway where the FAS–FASLG axis belongs to the later. p53 induces *FAS* expression when it binds to elements in the *FAS* gene promoter region or by recruiting FAS receptor from the Golgi apparatus of the cell for FASLG recognition^42^. Furthermore, p53 can sensitise cancer cells by upregulating their FAS receptor, to the apoptotic attack by cytotoxic T cells or NK-cells. These would recognise the cancer cell by binding its FAS receptor to the FASLG expressed on their membrane and hence induce apoptosis in a cancer cell^43^. Hence, mutation in the *TP53* gene will affect the apoptotic pathway of FAS–FASLG.

One limitation of this study is the rather small number of samples, which may have resulted in lost significance in some of the analyses, after performing multiple testing e.g. in the Ki-67 correlation test. In addition to FASLG, four other proteins were negatively correlated to Ki-67, including ANGPT1, NCR1, CXCL5, and TWEAK. These are proteins which are generally associated with angiogenesis, as well as cytotoxicity and induction of apoptosis^44–48^. Furthermore, six proteins (CCL4, CCL8, CCL23, CRTAM, CXCL1 and IL18) differed between the treatment responder and non-responder group, in an unadjusted way, but none of them were significant when corrected for multiple testing. A less prominent concentration of some proteins in patients with faster growing tumours, and difference between protein levels in treatment groups may prove to be of interest. These results may therefore warrant further investigation, preferably in larger materials.

In conclusion, we have identified proteins that may be of interest for further investigation but there was not one specific biomarker in which diagnostic or prognostic value could be placed on. The correlation of FASLG to Ki-67 and differentiation may be indicative of a possible new mechanism for FASLG in PanNENs G3 through an anti-tumour effect, although further studies are needed to confirm these findings. More studies in larger patient cohorts are needed to fully understand the FAS and FASLG pathways and their possible role in cancer progression or suppression.

## Materials and methods

### Patient characteristics

Blood (serum or plasma, based on availability) was collected from 42 PanNEN G3 patients from two Nordic centres and stored in − 80 °C until use. All patients had a PanNEN G3 diagnosed. All tumour specimens were positive for either one or both of the neuroendocrine biomarkers (CgA and Syn), and the median Ki-67 was 40% with missing data from two patients. The majority of patients were male (64%) and the median age was 59 years. Median overall survival was 16.5 months (range 0.6–65.3) with missing data from six patients. Blood (serum) from 42 healthy controls were matched to the best extent to patient’s characteristics regarding age and gender. We searched our pathology biobank for PanNEN G3 specimens and identified 16 samples available for immunohistochemistry. These were re-examined by an experienced pathologist (AP) with regards to differentiation.

All procedures performed in this study were in accordance with the ethical standards of the institutional and/or national research committee and with the 1964 Declaration of Helsinki and its later amendments, or comparable ethical standards. Informed consent was obtained from patients for participating in the registry and for biobanking of tumour tissue and blood samples. Ethical approval for the study was granted, and the need for new informed consent for this sub-study was waived for all participants by the ethical committee in Uppsala (2008/397/1, amendment approved 2017-08-24) and from the ethics committee in Norway.

### Proximity extension assay

The Olink Immuno-Oncology panel (Olink ONCOLOGY III, Olink Biosciences, Uppsala, Sweden) used in this study includes 92 proteins and for each sample 1 µL of blood was used. The PEA method is based on binding of PEA probes on paired antibodies, with affinity for each other. Binding of antibody to the target protein will bring the PEA probes closer together and DNA polymerisation starts with DNA polymerase. This results in a unique new DNA sequence which is a marker for the targeted protein. Detection of targeted proteins is obtained through quantitative real-time PCR^16^. The PCR results are analysed as Normalized Protein eXpression (NPX) values, which are arbitrary units on a log2-scale. NPX is calculated from Ct values and data pre-processing is performed to minimize both intra- and inter-assay variation. A high NPX value corresponds to a high protein concentration and expresses relative quantification between samples but represents no absolute quantification. Assay characteristics including quality control, detection limits and measurements of assay performance and validation can be obtained via Olink’s website (https://www.olink.com). The analysis was performed by Olink Biosciences, Uppsala, Sweden.

### Immunohistochemistry

Whole-section tumour samples from 16 PanNEN G3 patients with available paraffin-embedded tissue material were collected and cut into 4 µm sections. Sections were deparaffinised in xylene, hydrated in graded alcohols and blocked for endogenous peroxidase in 0.3% hydrogen peroxide diluted in 95% ethanol. For antigen retrieval, a Decloaking Chamber (Biocare Medical, Pacheco, CA) was used. Slides were immersed and boiled in citrate buffer, pH 6 (Lab Vision, Fremont, CA) for 4 min at 125 °C and then allowed to cool to 90 °C (the total program is approximately 40 min). Automated immunohistochemistry was performed essentially as previously described^49,50^ using an Autostainer 480 instrument (Thermo Fisher Scientific, Waltham, MA). The primary rabbit polyclonal antibody against FAS ligand (FASLG) (HPA054959, Atlas Antibodies, Stockholm, Sweden) was diluted in 1:800 UltraAb Diluent (Thermo Fisher Scientific) followed by incubation for 30 min at room temperature (RT). The slides were further incubated with the secondary reagent anti-rabbit/mouse horseradish peroxidase-conjugated UltraVision (Thermo Fisher Scientific) for 30 min at RT, and developed for 10 min using Diaminobenzidine (DAB) Quanto (Thermo Fisher Scientific) as chromogen. All incubations were followed by rinse in wash buffer (Thermo Fisher Scientific) 2 × 5 min. Slides were counterstained in Mayer’s Hematoxylin (Histolab, Gothenburg, Sweden) and cover slipped using Pertex (Histolab) as mounting medium. Human tonsil was used as a positive control.

The analysis of the FASLG immunostainings was blindly performed by one pathologist (AP), followed by three independent observers (AA, MG and ETJ). H-score was calculated for each sample. The intensity of FASLG staining was graded 0 for negative, 1 for intermediate and 2 for strong, and this was multiplied by the percentage of stained tumour cells. Consistently weak positive FASLG staining was present in the normal pancreatic islets.

### Statistical analysis

Statistical analysis was performed by the Olink Biostatistics unit. Four different statistical methods were used. *T*-test was used to identify differences in protein concentrations between healthy controls and tumour patients. Regression analysis was conducted to search for correlation between protein concentrations and Ki-67 index in tumour patients. Survival analysis was used to investigate associations between protein concentration and survival in tumour patients. Finally, a one-way ANOVA test was performed to compare protein concentrations in different treatment response groups. All *p* values were adjusted for multiple testing within each test using the Benjamini–Hochberg approach^51^.

A chi-square test was performed to study correlations between FASLG expression and tumour differentiation (IBM SPSS statistics software, v25, USA).

## Data Availability

The datasets generated during and/or analysed during the current study are available from the corresponding author on reasonable request.
